# Supramolecular combination chemotherapy: a pH-responsive co-encapsulation drug delivery system†
†Electronic supplementary information (ESI) available. See DOI: 10.1039/d0sc01756f


**DOI:** 10.1039/d0sc01756f

**Published:** 2020-06-03

**Authors:** Junyi Chen, Yadan Zhang, Zhao Meng, Lei Guo, Xingyi Yuan, Yahan Zhang, Yao Chai, Jonathan L. Sessler, Qingbin Meng, Chunju Li

**Affiliations:** a State Key Laboratory of Toxicology and Medical Countermeasures , Beijing Institute of Pharmacology and Toxicology , Beijing 100850 , P. R. China . Email: nankaimqb@sina.com; b Department of Chemistry , Center for Supramolecular Chemistry and Catalysis , Shanghai University , Shanghai 200444 , P. R. China . Email: sessler@cm.utexas.edu; c Key Laboratory of Inorganic–Organic Hybrid Functional Material Chemistry , Ministry of Education , Tianjin Key Laboratory of Structure and Performance for Functional Molecules , College of Chemistry , Tianjin Normal University , Tianjin 300387 , P. R. China . Email: cjli@shu.edu.cn; d Key Laboratory of Advanced Energy Materials Chemistry (Ministry of Education) , College of Chemistry , Nankai University , Tianjin , 300071 , China; e Key Laboratory of Natural Resources and Functional Molecules of the Changbai Mountain , Affiliated Ministry of Education , College of Pharmacy , Yanbian University , Yanji , Jilin , 133002 , China

## Abstract

Most cancer chemotherapy regimens rely on the use of two or more chemotherapeutic agents. However, achieving the best possible dosing of the individual drugs can be challenging due to differences in metabolism, uptake, and clearance among other factors. Here we describe a supramolecular strategy for achieving drug delivery in which the loading ratio of two active components is easily defined. Specifically, we report the formation of aggregates comprised of self-assembled amphiphiles between carboxylatopillar[6]arene (CP6A) and an oxaliplatin (OX)-type Pt(iv) prodrug (PtC_10_). The association constant (*K*_a_) for the underlying host–guest interaction at pH 7.4 ((1.16 ± 0.03) × 10^4^ M^–1^) is an order of magnitude higher than at pH 5.0 ((1.73 ± 0.15) × 10^3^ M^–1^). A second chemotherapeutic, doxorubicin (DOX), may be encapsulated in the resulting vesicles (PtC_10_⊂CP6A) to give a supramolecular combination chemotherapeutic system DOX@PtC_10_⊂CP6A. Drug release studies served to confirm that PtC_10_ and DOX are released in acidic environments. Support for a synergistic antiproliferative effect relative to PtC_10_ + DOX came from cellular studies of DOX@PtC_10_⊂CP6A using the human liver hepatocellular carcinoma (HepG-2) cell line. *In vivo* studies revealed that DOX@PtC_10_⊂CP6A is not only able to retard tumor growth efficiently but also reduce drug-related toxic side effects in BALB/c nude mice bearing HepG-2 subcutaneous tumor xenografts. These favorable findings are attributed to the formation of a ternary complex that benefits from an enhanced permeability and retention (EPR) effect *in vivo* while allowing for the pH-based release of PtC_10_ and DOX at the tumor site.

## Abstract 2

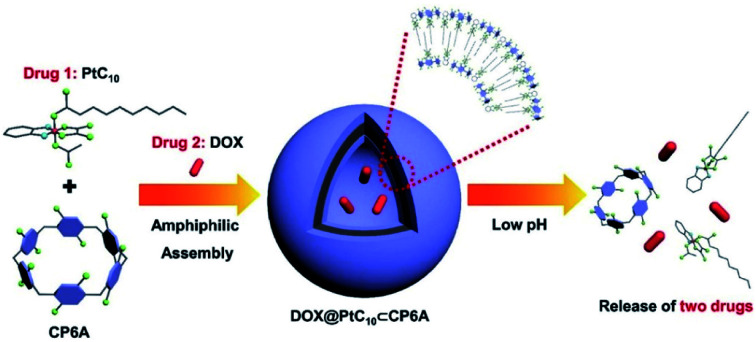
Most cancer chemotherapy regimens rely on the use of two or more chemotherapeutic agents. A supramolecular approach that may allow co-delivery of two drugs is described here.

## Introduction

Clinical management of malignant tumors typically relies on the use of more than one chemotherapeutic agent. This is because single-agent chemotherapy can induce tumor proliferation and or trigger hard-to-overcome resistance mechanisms. This can lead to multidrug resistance and tumor recurrence.^1^,^2^ Thus, combination chemotherapeutic protocols are the norm.^3^ However, on account of the different pharmacokinetics of each drug within a given treatment cocktail, it is often difficult to control the constituent drug distribution and tumor-specific concentrations *in vivo*. Moreover, different drug ratios can lead to either synergistic, additive, or antagonistic effects, resulting in uncertain clinical outcomes.^4^–^6^ Advanced drug delivery systems (DDSs) with an ability to encapsulate multiple agents simultaneously and deliver them concurrently to tumors may overcome these recognized limitations.^7^,^8^

DDS currently being used for combination chemotherapy include liposomes,^9^–^11^ polymeric nanoparticles,^12^–^14^ organic–inorganic hybrid materials,^15^–^17^ among other approaches.^18^–^20^ As detailed below, we propose a novel supramolecular combination chemotherapy system that permits the co-delivery of two recognized chemotherapeutics, namely oxaliplatin (OX) (in the form of a Pt(iv) prodrug) and doxorubicin (DOX). The present strategy is attractive in that drug release is triggered efficiently as the result of pH responsive host–guest interactions.^21^–^26^

OX, a diaminocyclohexane analogue of cisplatin, constitutes the third platinum drug approved by the US FDA.^27^–^29^ In spite of its better tolerability compared to other Pt(ii) compounds, including cisplatin and carboplatin, OX still suffers from low selectivity and dose-limiting side effects.^30^–^32^ Nevertheless, OX in combination with fluorouracil,^33^,^34^ capecitabine,^35^,^36^ doxorubicin^37^,^38^ and other agents,^39^,^40^ is either used clinically or the subject of ongoing clinical studies. For example, OX and DOX are used in combination for the management of patients suffering from hepatocellular carcinoma.^41^,^42^ Although not yet benefiting from FDA approval, Pt(iv) complexes have attracted attention recently as potential Pt(ii) prodrugs. As a generally rule, Pt(iv) species are less reactive (labile) than the corresponding Pt(ii) congeners, which reduces concerns involving systemic toxicity. These prodrugs are thought to undergo reduction to release an active platinum(ii) species that then mediates an antitumor cytotoxic effect through *inter alia* binding to DNA.^43^–^45^

**Scheme 1:**
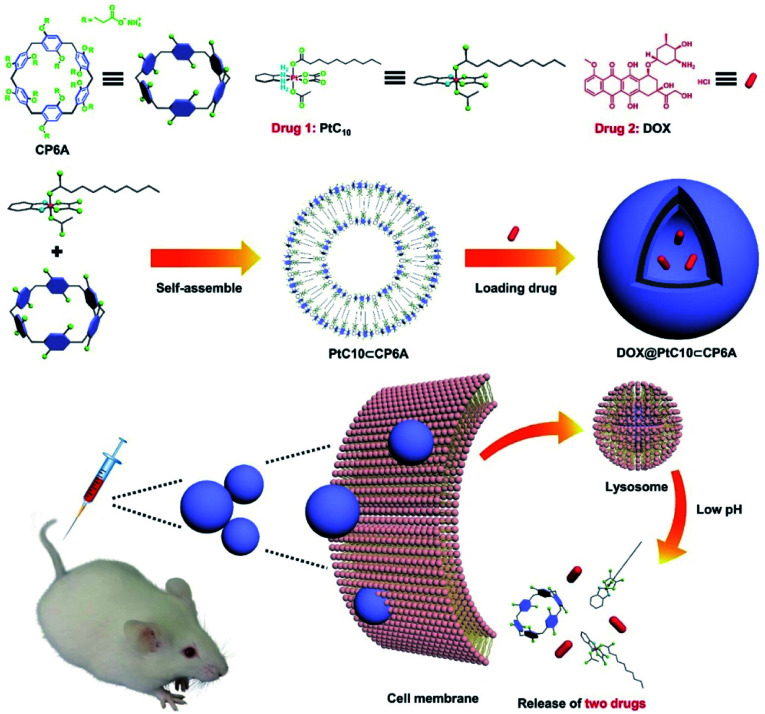
Chemical structures of CP6A and PtC_10_, schematic illustration of the preparation of DOX@PtC_10_⊂CP6A, and the proposed mechanism for drug release.

In the present study, a supramolecular amphiphilic complex derived from carboxylatopillar[6]arene (CP6A) and an OX-based Pt(iv) prodrug (PtC_10_) were used to construct nano-scale aggregates that encapsulate DOX effectively. The resulting supramolecular combination chemotherapy system (DOX@PtC_10_⊂CP6A) was found to target tumor tissues passively through an enhanced permeability and retention (EPR) effect. However, they were then seen to collapse in the lower pH lysosomal environment after cellular uptake to release the two drugs, PtC_10_ and DOX (Scheme 1). Evidence for a synergistic antitumor effect was then seen. This work thus serves to highlight the inherent promise of smart supramolecular self-assembled amphiphiles as DDS for combination chemotherapy.

## Results and discussion

### Host–guest complexation studies

**Fig. 1:**
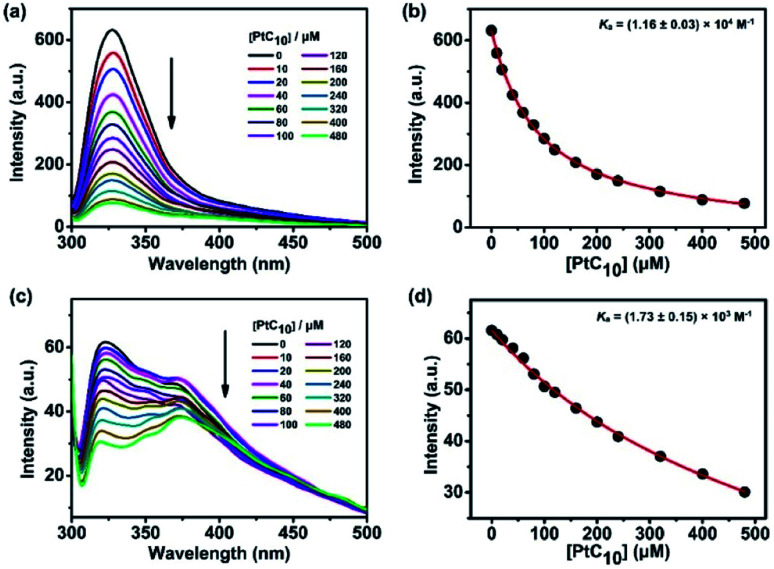
Fluorescence spectra of CP6A (1.0 × 10^–5^ M) in aqueous PBS (a) pH 7.4 and (c) pH 5.0 recorded in the presence of different concentrations of PtC_10_ at 298 K. (b) and (d) nonlinear least-squares analyses used to calculate the *K*_a_ value.

CP6A, a readily accessible water-soluble derivative of pillararene (PA),^46^ is an effective host for oxaliplatin (OX).^47^ As reported previously, CP6A has a diameter of *ca.* 6.7 Å.^48^ It thus matches well the cyclohexyl moiety of OX in terms of both size and shape. Pt(iv) complexes typically carry two additional ligands as compared to the corresponding Pt(ii) species. These ancillary ligands can be used to introduce hydrophobic groups that can facilitate the construction of PA-based self-assembled amphiphiles.^46^ With such thinking in mind, we prepared PtC_10_, a prodrug of OX analogous to one first reported by Ammar, *et al.* (Fig. S1†).^49^ PtC_10_ is less water soluble than OX, presumably due to the presence of the hydrophobic alkyl chain. Because of this reduced water solubility, the host–guest complexation interactions between CP6A and PtC_10_ could not be investigated directly by NMR spectroscopy in D_2_O. As a consequence, the host–guest association constants were determined at lower concentration by means of fluorescence titration experiments carried out in aqueous phosphate buffered saline (PBS) at both pH 7.4 and 5.0. Continuous variation method (Job's plot method) results proved consistent with a 1 : 1 binding stoichiometry between the host and guest (Fig. S6 and S7†). As shown in Fig. 1, CP6A binds PtC_10_ strongly at pH 7.4, with a *K*_a_ value of (1.2 ± 0.03) × 10^4^ M^–1^ being determined *via* standard curve fitting protocols. This *K*_a_ value is an order of magnitude higher than what is seen at pH 5.0 (*K*_a_ = (1.7 ± 0.2) × 10^3^ M^–1^). The substrate-induced spectral changes were also less clean in this latter pH regime. This pH dependence is ascribed to the structure of CP6A, which bears 12 weakly basic carboxylate moieties that are expected to exist largely in their anionic (deprotonated) forms at pH 7.4. This allows for stabilizing electrostatic interactions with the positively charged PtC_10_ guest. Under acidic conditions, these carboxylate moieties are partially protonated, reducing the binding affinity. The pH values 7.4 and 5.0 reflect those of normal physiological environment and lysosomes, respectively. We thus postulated that the host–guest complex, PtC_10_⊂CP6A, produced *via* self-assembly would most likely show pH responsive behavior and disassociate within lysosomes or in acidic tumor microenvironments.

### Supramolecular vesicle assembly

When the solutions produced by mixing CP6A and PtC_10_ in double-distilled water (pH = 6.8) were allowed to sit for 0.5 h, a red opalescence was observed, the intensity of which varied with the [CP6A]/[PtC_10_] ratio. This was taken as evidence of aggregate formation. Pyrene was used as a fluorescent probe to study these relative concentration effects. Pyrene is expected to be bound by the micelle-like species produced *via* aggregation and display minimal emission intensity in such a bound state. Based on experiments where the relative ratio of CP6A and PtC_10_ were varied, the lowest intensity was seen at [CP6A]/[PtC_10_] = 1 : 2 (Fig. S9a and b†). This finding leads us to infer that this ratio best favors formation of PtC_10_⊂CP6A aggregates. With [CP6A]/[PtC_10_] = 1 : 2, the critical aggregation concentration (CAC) for PtC_10_⊂CP6A was determined to be 12.5 μM (for PtC_10_), while free PtC_10_ or CP6A did not tend to form aggregates even at 200 μM (Fig. S10a and b†).

**Fig. 2:**
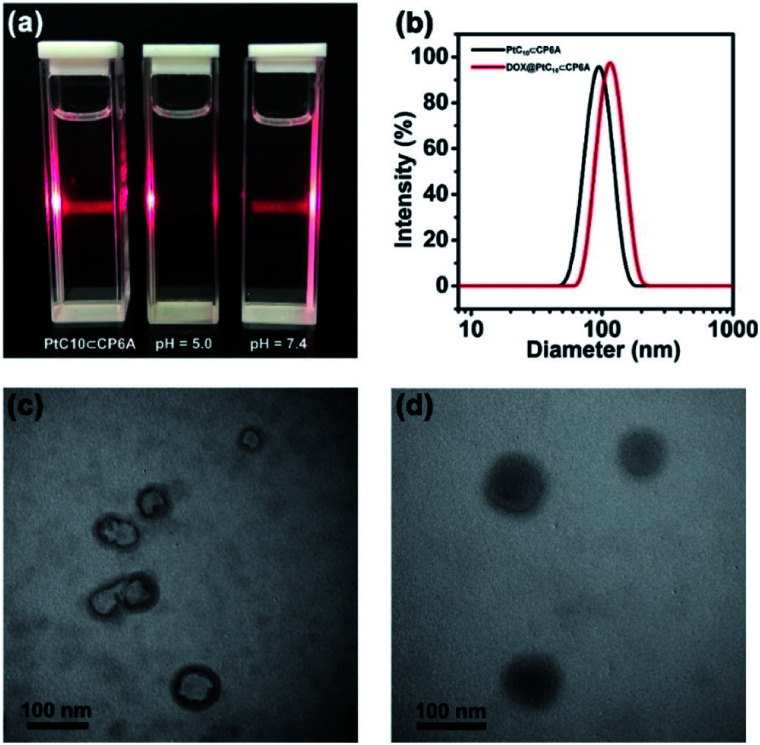
(a) Tyndall effect seen for PtC_10_⊂CP6A aggregates prepared in aqueous solutions of different pH. (b) DLS data for PtC_10_⊂CP6A and DOX@PtC_10_⊂CP6A (298 K, scattering angle = 90°). TEM images: vesicles self-assembled from (c) PtC_10_⊂CP6A and (d) DOX@PtC_10_⊂CP6A ([CP6A] = 0.05 mM and [PtC_10_] = 0.10 mM).

Aqueous mixtures of PtC_10_⊂CP6A produced using [CP6A]/[PtC_10_] = 1 : 2 per the above, exhibited a notable Tyndall effect (Fig. 2a). Such a finding provides support for the existence of nanoparticles. Transmission electron microscopy (TEM) images proved consistent with the formation of hollow supramolecular vesicles with diameters ranging from 50 nm to 90 nm (Fig. 2c and S11a†). Results obtained from dynamic laser scattering (DLS) measurements were consistent with an average particle size of 91.3 nm (Fig. 2b). The thickness of the outer wall of the PtC_10_⊂CP6A nanoparticle was about 6 nm, as inferred from TEM studies (Fig. 2c). This value is consistent with the bilayer molecular length of PtC_10_⊂CP6A simulated by Chem3D (Fig. S12†). The resulting supramolecular vesicles were predicted to possess a bilayer structure with two hydrophilic carboxylate shell layers, as well as a core layer containing the hydrophobic alkyl chains. This prediction reflects an appreciation that the cyclohexyl group present in PtC_10_ would be bound within the cavity of the CP6A receptor as the result of solvatophobic interactions. The CP6A moiety would then serve as a hydrophilic head group, while the ancillary alkyl ligands would serve as hydrophobic tails. The net result is a set of tadpole-like self-assembled amphiphiles (Scheme 1). The zeta potential of PtC_10_⊂CP6A was determined to be –30.6 mV (Fig. S13†), leading us to suggest that electrostatic repulsion could facilitate the stabilization of supramolecular vesicles when CP6A is combined with PtC_10_ at pH = 7.4.

As noted above, lowering the pH from 7.4 to 5.0 served to decrease the interaction between PtC_10_ and CP6A. To probe whether the resulting PtC_10_⊂CP6A vesicles also displayed pH responsiveness, the solution pH was adjusted to 5.0. This led to a loss of the Tyndall effect, which reappeared when the pH was readjusted back to 7.4 (Fig. 2a). This switching is taken as evidence that mixing PtC_10_ and CP6A at a pH of 7.4 followed by time-dependent aggregation leads to formation of pH-responsive supramolecular vesicles.

### Encapsulation of DOX and *in vitro* drug release

As supported by the TEM and light scattering studies, the PtC_10_⊂CP6A supramolecular vesicles prepared in double distilled water are expected to possess hydrophilic interiors. To the extent this supposition is correct, we envisioned that PtC_10_⊂CP6A could be used as a nano-scale carrier capable of encapsulating another hydrophilic anti-tumor drug, thus allowing co-delivery. In clinical practice, OX is often used in combination with DOX as noted above.^43^–^45^ Therefore, DOX was used to test whether PtC_10_⊂CP6A would form a supramolecular construct that could be used as a dual agent DDS.

Adding DOX to an aqueous solution of PtC_10_⊂CP6A led to a change from colorless to light red (Fig. S14†). This was taken as a preliminary indication that DOX was successfully encapsulated into PtC_10_⊂CP6A vesicles to form a two-drug construct DOX@PtC_10_⊂CP6A. Incorporation of DOX into vesicles was accompanied by changes in the zeta potential. Specifically, after loading, the zeta potential decreased from –30.6 to –27.3 mV, an effect ascribed to uptake of the positively charged DOX (Fig. S15†). Changes in the morphology and size distribution of the presumed DOX@PtC_10_⊂CP6A DDS were observed, as inferred from TEM and DLS measurements. As shown in Fig. 2b, d and S10b,† upon treatment with DOX the hollow vesicles ascribed to PtC_10_⊂CP6A were replaced by *ca.* 100 nm diameter nanoparticles with dark interiors. DLS studies gave an average diameter of 122 nm for the presumed DOX@PtC_10_⊂CP6A constructs. Importantly, no change in the size of the DOX@PtC_10_⊂CP6A ensembles was seen when they were allowed to stand in double-distilled water for 3 days (Fig. S16†); this was taken as evidence of their high stability, at least under these conditions.

The size range for DOX@PtC_10_⊂CP6A was considered to augur well for potential biological use. Previous studies have shown that particles in the range of tens to hundreds nanometers often display favorable pharmacokinetic characteristics. For instance, they typically accumulate within tumor tissues as the result of an EPR effect; this, in turn, can increase the therapeutic efficiency and reduce the toxic side effects of nanoparticle-based treatment protocols.^9^–^11^ Accordingly, efforts were made to explore further whether DOX@PtC_10_⊂CP6A could be used to effect the co-delivery of the two bound drugs (DOX and PtC_10_) to cancer cells *in vitro* and to tumor targets *in vivo*.

High-performance liquid chromatography (HPLC) was used to determine accurately the concentration of the two drug components present in DOX@PtC_10_⊂CP6A. First, appropriate calibration curves were derived (Fig. S17a and b†). Using these curves and the peak area obtained from diluted samples, the encapsulation efficiency of PtC_10_ and DOX was calculated to be 83.8% and 25.8%, respectively. This corresponds to a molar ratio of 3.25. Fortuitously, this matches well the relative dosages in clinical use, namely OX = 130 mg m^–2^ and DOX = 60 mg m^–2^ or a molar ratio of 3.17. Synergy effects matching those seen for OX + DOX were thus expected for DOX@PtC_10_⊂CP6A.

Prior to carrying out biological tests with DOX@PtC_10_⊂CP6A, we sought to test whether OX and DOX would be released as the pH was lowered. As noted above, the carboxylate moieties of CP6A are partially protonated under acidic conditions. This leads to a weakening of the interaction between CP6A and PtC_10._ and effective release of the two components (DOX and PtC_10_).

**Fig. 3:**
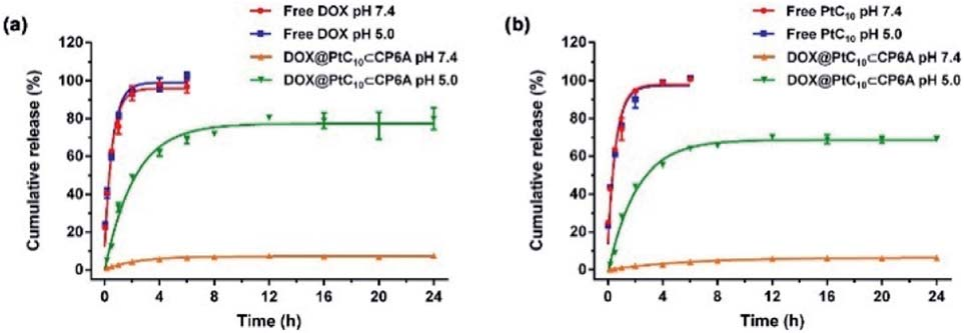
Time dependent release of (a) DOX and (b) PtC_10_ from DOX@PtC_10_⊂CP6A in PBS at different pH (mean ± SD, *n* = 3). Concentrations at any given time point were determined by HPLC. See the ESI for details.†

It is well known that lysosomes are acidic organelles, typically characterized by a pH of 5.0 or lower.^50^,^51^ The drug release behavior of DOX@PtC_10_⊂CP6A was thus investigated at pH 5.0. As shown in Fig. 3a, approximately 7% of the bound DOX was released from DOX@PtC_10_⊂CP6A over the course of 24 h when this construct was placed in a dialysis bag at pH 7.4 and allowed to equilibrate. In contrast, a cumulative release of about 80% within 24 h was seen at pH 5.0. Similar release behavior was seen for PtC_10_; at pH 7.4 the cumulative release of PtC_10_ was only about 6% over the course of 24 h but about 70% at a pH of 5.0 under otherwise identical conditions (Fig. 3b).

Fluorescence spectroscopy was used to investigate further the release behavior (Fig. S18†). No fluorescence signal could be observed for aqueous pH 7.4 mixtures of PtC_10_⊂CP6A over the spectral range corresponding to the DOX-based emission. A weak fluorescence signal was seen for DOX@PtC_10_⊂CP6A, a finding ascribed to the encapsulation of DOX. Upon adjusting the solution pH to 5.0, the fluorescence intensity of DOX was enhanced. This increase is taken as evidence that DOX@PtC_10_⊂CP6A undergoes pH-dependent vesicle collapse with concomitant release of DOX. In control studies, DOX@PtC_10_⊂CP6A was treated with Triton X-100 so as to achieve the complete release of DOX thus allowing comparisons with the release thought to be triggered by lowering the pH. Taken together, these results lead us to suggest that DOX@PtC_10_⊂CP6A may have a role to play as a pH-responsive co-delivery system.

### *In vitro* cytotoxicity and cellular uptake

**Fig. 4:**
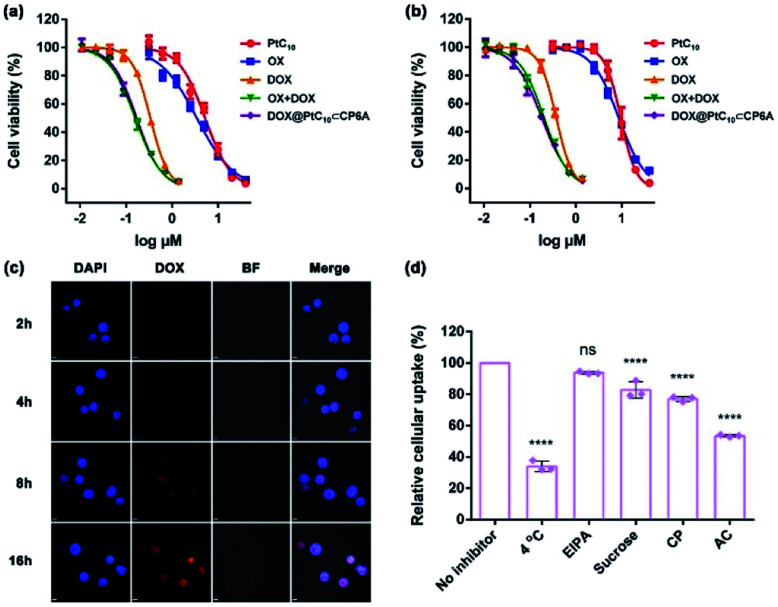
Cytotoxicity seen in the (a) HepG-2 and (b) LO2 cell lines incubated with the indicated agents for 72 h. Cell death was measured using CCK-8 assays (mean ± SD, *n* = 5). (c) Cellular uptake and intracellular location studies using HepG-2 cells incubated with DOX@PtC_10_⊂CP6A (containing 10 μM DOX) for approximately 2, 4, 8 and 16 h, respectively. Cell nuclei were stained by DAPI (scale bar: 20 μm). BF = bright field. DIPA = 4′,6-diamidino-2-phenylindole. Cell uptake was determined by confocal laser scanning microscopy. (d) Effect of different inhibitors on the internalization of DOX@PtC_10_⊂CP6A (containing 10 μM DOX) into HepG-2 cells after a 4 h incubation period at 37 °C unless otherwise indicated, as determined by flow cytometry (mean ± SD, *n* = 3). EIPA = 5-(*N*-ethyl-*N*-isopropyl) amiloride, CP = chlorpromazine, and AC = ammonium chloride. The significance of the differences seen in (d) were assessed using one-way ANOVA tests and multiple comparisons; ns = not significant; ****P < 0.0001.

To examine the effect of DOX@PtC_10_⊂CP6A on cell viability, the cytotoxicity of CP6A in the human liver hepatocellular carcinoma (HepG-2) and human normal liver (LO2) cell lines was assessed using a Cell Counting Kit-8 (CCK-8) assay. On the basis of these studies and prior work,^48^ we conclude that even at relatively high concentrations CP6A is relatively nontoxic (Fig. S19†). These same HepG-2 and LO2 cell lines were then used to test the *in vitro* cell inhibitory effect of DOX@PtC_10_⊂CP6A in conjunction with various positive controls (Fig. 4a and b). Concentration-dependent cell death was observed for all formulations and the half-maximum inhibitory concentration (IC_50_) values for a 72 h incubation period were then determined and the combination index (CI) calculated (Table S1†). Two drugs are considered to be synergistic when the CI value is less than 1.00. The cytotoxicity of PtC_10_ proved to be slightly lower than that of OX both in the HepG-2 and LO2 cell lines. The IC_50_ of DOX for HepG-2 cell line (0.34 μM) matched that recorded in the literature.^52^,^53^ The CI value of OX + DOX in the HepG-2 cell line was 0.62, indicating that in our hands the combination of OX and DOX exhibits a synergistic effect at the cellular level. The CI value of DOX@PtC_10_⊂CP6A (0.61) was found to match that of OX + DOX, leading us to infer that this self-assembled DDS system promotes a synergistic effect *in vitro*. Similar findings were found using the LO2 cell line.

The cellular uptake and intracellular drug release features of DOX@PtC_10_⊂CP6A were then investigated using confocal laser scanning microscopy (CLSM) and the HepG-2 cell line. 4′,6-Diamidino-2-phenylindole (DAPI) was used to stain the cell nucleus blue. Upon incubation with DOX@PtC_10_⊂CP6A for 2 h, a weak red fluorescence ascribed to DOX was observed in HepG-2 cells (Fig. 4c). These red dots were largely colocalized with DAPI. Moreover, the fluorescence intensity increased as the incubation time increased. This is as expected for a DDS system that releases DOX in a time-dependent manner. The presumed cellular internalization was further studied using flow cytometry (Fig. 4d and S20†). The uptake of DOX@PtC_10_⊂CP6A in HepG-2 cells was reduced to less than 35% upon incubation at 4 °C, as measured by flow cytometry. This finding lends credence to the conclusion that the observed internalization is mediated primarily by an energy-dependent endocytosis process. Negligible uptake inhibition was seen upon co-incubation with 5-(*N*-ethyl-*N*-isopropyl) amiloride (EIPA), a macropinocytosis inhibitor. This leads us to suggest that there is minimal involvement of this potential uptake pathway.

We also investigated the effect of sucrose and chlorpromazine (CP). Both agents act as inhibitors of clathrin-coated vesicle formation. Previous studies have served to confirm that nanoparticles between 100 and 200 nm in diameter, the size of DOX@PtC_10_⊂CP6A, are subject to clathrin-mediated endocytosis.^54^,^55^ It was found that the cellular uptake of DOX@PtC_10_⊂CP6A as inferred from flow cytometry was reduced in a statistically meaningful way in the presence of sucrose or CP. The effect of the lysosome function inhibitor, ammonium chloride (AC), was also tested. It was found that the uptake of DOX@PtC_10_⊂CP6A into HepG-2 cells was inhibited by about 50% in the presence of AC. This result was taken as evidence that DOX@PtC_10_⊂CP6A enters HepG-2 cells in part through the lysosomes.

### *In vivo* tumor inhibition studies

**Fig. 5:**
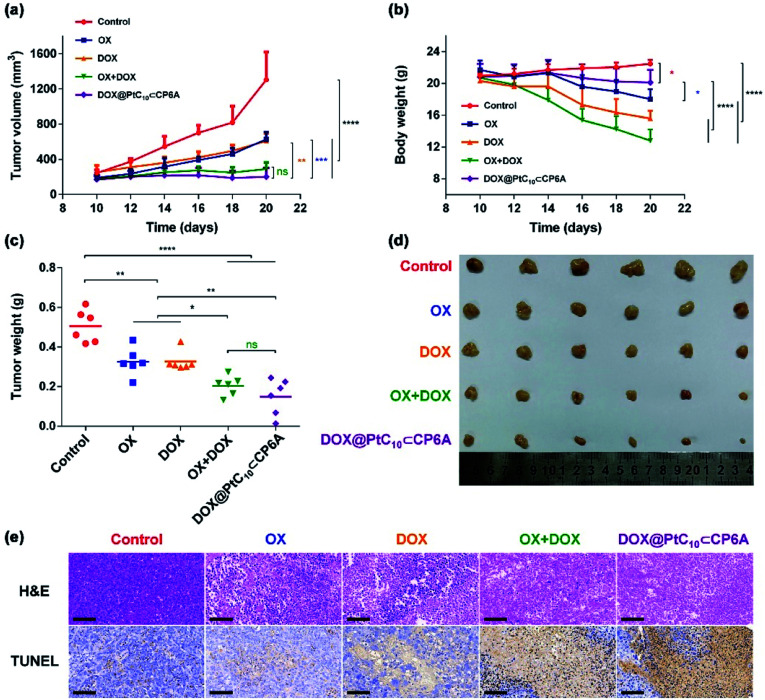
*In vivo* antitumor experiments. (a) Tumor growth curves for HepG-2 xenograft BALB/c nude mice treated with 5% glucose solution (control), OX, DOX, OX + DOX, and DOX@PtC_10_⊂CP6A (mean ± SD, *n* = 6). (b) Body weight changes observed for HepG-2 xenograft nude mice (mean ± SD, *n* = 6). (c) Average tumor weight determined 10 days after treatment with 5% glucose solution (control), OX, DOX, OX + DOX, or DOX@PtC_10_⊂CP6A (mean ± SD, *n* = 6). (d) Images of tumors excised from HepG-2 xenograft nude mice treated with 5% glucose solution (control), OX, DOX, OX + DOX, or DOX@PtC_10_⊂CP6A. (e) H&E and TUNEL analyses of tumor tissue excised after the indicated treatments (scale bar: 100 μm). Significant differences were assessed in (a), (b), and (c) using the two-way ANOVA with multiple comparisons. ns, not significant. *P < 0.05, **P < 0.01, ***P < 0.001, ****P < 0.0001.

HepG-2 derived subcutaneous tumor xenograft mouse models were used to test the anti-tumor efficacy of DOX@PtC_10_⊂CP6A *in vivo*. OX, DOX, OX + DOX, and 5% glucose were tested as controls. Note: OX, rather than free PtC_10_, was used for these control studies owing to the poor water solubility of the latter Pt(iv) complex. An *n* = 6 was used for each group. The tumor volumes of mice administrated a 5% glucose solution (negative control) were found to increase by more than five-fold over the course of 10 days, while various degrees of tumor growth inhibition was seen for the other groups (Fig. 5a and d). Compared to the glucose control group, OX and DOX resulted in a 52% and 53% inhibition of tumor growth, respectively. Treatment with OX + DOX and DOX@PtC_10_⊂CP6A led to more effective tumor growth inhibition (*i.e.*, 78% and 85%, respectively), consistent with the synergistic effects inferred from the *in vitro* studies.

The normalized tumor weight was assessed for the various groups (Fig. 5c). The average tumor weight of the glucose control group (0.51 ± 0.08 g) was 55% higher than that of the OX group (0.32 ± 0.07 g) or DOX group (0.32 ± 0.05 g). The tumor weight of the OX + DOX and DOX@PtC_10_⊂CP6A groups were 0.20 ± 0.05 g and 0.15 ± 0.09 g, respectively. Thus, a large and statistically significant reduction in tumor regrown was seen for these two group relative to the control or OX and DOX alone.

Immunohistochemical analyses, including hematoxylin and eosin (H&E) staining and terminal deoxynucleotidyl transferase dUTP nick end labeling (TUNEL) assays, were used to assess the anti-tumor efficiency of the various groups (Fig. 5e). Imaging of H&E-stained tumor tissue from the control group revealed spindle shapes and unbroken nuclei, features that are characteristic of rapid tumor growth. As compared to the control group, images from both the OX + DOX and DOX@PtC_10_⊂CP6A groups revealed a loss of nuclei. Furthermore, treatment with either OX + DOX or DOX@PtC_10_⊂CP6A was found to induce a greater level of TUNEL-positive cells. These results were taken as further evidence that both free OX + DOX or DOX@PtC_10_⊂CP6A were effective at mediating an antitumor response.

In clinical use, both OX and DOX can induce a number of toxicity-related side reactions, including nausea, vomiting, thrombocytopenia, leukopenia, and in the case of DOX, cardiotoxicity. In the present study, the change in body weight of tumor-bearing mice after administration was taken as a surrogate for acute systematic toxicity. As shown in Fig. 5b, mice treated with free OX suffered a barely significant body weight loss from 21.7 ± 1.2 to 18.0 ± 1.2 g. The body weight of the DOX group decreased from 20.3 ± 0.8 to 15.6 ± 1.0 g. Mice administrated OX + DOX suffered a body weight loss from 20.7 ± 1.2 to 12.8 ± 1.4 g. In contrast, almost no body weight changes were observed for the mice treated with DOX@PtC_10_⊂CP6A (non-statistically significant decrease from 20.8 ± 1.6 to 20.1 ± 1.6 g). These favorable findings were taken as support for the proposition that DOX@PtC_10_⊂CP6A acts as a supramolecular DDS system and enhances the free drug concentration at the tumor tissue as the result of an EPR effect and site specific release of OX and DOX. In normal biological environments a large *K*_a_ is expected to favor PtC_10_⊂CP6A formation and preclude substantial drug loss or leakage. However, in the acidic environment of lysosomes and solid tumors, dissociation of PtC_10_⊂CP6A and release of DOX and PtC_10_ is expected to occur. The above whole animal studies are fully consistent with this design expectation.

Further support for the low toxicity inferred for DOX@PtC_10_⊂CP6A came from histological analyses of major organ slices, including those of the heart, liver, spleen, lung and kidney of the mice used in the above studies (Fig. S21†). Compared with the control group, severe splenic toxicity and notable cardiotoxicity was observed in the OX + DOX group. Evidence of characteristic inflammation and necrosis in splenocytes and cytoplasmic relaxation in cardiomyocytes was also seen. In contrast, treatment with DOX@PtC_10_⊂CP6A induced much lower organ toxicity as inferred from the corresponding histological analyses. On this basis, we propose that the supramolecular DDS combination chemotherapy strategy embodied in DOX@PtC_10_⊂CP6A can be used decrease the undesirable side effects of OX and DOX while maintaining good antitumor efficacy. To the extent this favorable augury translates into the clinic, it is expected to provide a significant and salutary benefit for patients.

## Conclusions

In summary, we have successfully prepared a pH-responsive co-delivery system that provides for combination chemotherapy. This system consists of supramolecular amphiphilic complex PtC_10_⊂CP6A and DOX. PtC_10_⊂CP6A itself is prepared from PtC_10_ and CP6A. Once formed, PtC_10_⊂CP6A self-assembles in neutral aqueous media to produce hollow vesicles with an average diameter of ∼90 nm. These vesicles possess a hydrophilic cavity that can encapsulate DOX to produce a pH-responsive co-delivery system containing two drug components. Drug release experiments provided support for the expectation that both the PtC_10_ and DOX species are released efficiently at pH 5.0. It was also found that DOX@PtC_10_⊂CP6A could enter HepG-2 cells, predominantly through endocytosis, and that the DOX presumably released *in vitro* from DOX@PtC_10_⊂CP6A would mark the cell nucleus. DOX@PtC_10_⊂CP6A provided for a synergistic cell killing effect compared to either PtC_10_ or DOX as determined using the HepG-2 and LO2 cell lines *in vitro*. *In vivo* anti-tumor experiments demonstrated that DOX@PtC_10_⊂CP6A exhibited higher therapeutic efficiency and engendered less body weight loss in HepG-2 tumor xenograft bearing nude mice as compared to various controls. This work thus serves to highlight what may emerge as an effective and readily generalizable strategy for improving combination chemotherapy. To the extent this proves true, it could lead in due course to potential clinical treatment benefits.

## Conflicts of interest

There are no conflicts to declare.

## Supplementary Material

Supplementary information
