# Supplementary material for: Supramolecular combination chemotherapy: a pH-responsive co-encapsulation drug delivery system
Source: Chem Sci. 2020 Jun 3;11(24):6275–82. doi: 10.1039/d0sc01756f (PMC7473403; doi:10.1039/d0sc01756f)
Supplement: Supplementary file 1 [file SC-011-D0SC01756F-s001.pdf]

Electronic Supplementary Material (ESI) for Chemical Science.
This journal is © The Royal Society of Chemistry 2020

Electronic Supplementary Material (ESI) for Chemical Science.
This journal is © The Royal Society of Chemistry 2020

Supporting Information for

Supramolecular Combination Chemotherapy: A pH-Responsive Co-Encapsulation Drug Delivery System

Junyi Chen,[a,b] Yadan Zhang,[a] Zhao Meng,[a] Lei Guo,[a] Xingyi Yuan,[a,b] Yahan Zhang,[a] Yao Chai,[a,b] Jonathan L. Sessler,*[b] Qingbin Meng,*[a,d,e] and Chunju Li*[b,c]

[a]State Key Laboratory of Toxicology and Medical Countermeasures, Beijing Institute of Pharmacology and Toxicology, Beijing 100850, P. R. China.
[b]Department of Chemistry, Center for Supramolecular Chemistry and Catalysis, Shanghai University, Shanghai 200444, P. R. China.
[c]Key Laboratory of Inorganic-Organic Hybrid Functional Material Chemistry, Ministry of Education, Tianjin Key Laboratory of Structure and Performance for Functional Molecules, College of Chemistry, Tianjin Normal University, Tianjin 300387, P. R. China.
[d]Key Laboratory of Advanced Energy Materials Chemistry (Ministry of Education), College of Chemistry, Nankai University, Tianjin, 300071, China.
[e]Key Laboratory of Natural Resources and Functional Molecules of the Changbai Mountain, Affiliated Ministry of Education, College of Pharmacy, Yanbian University, Yanji, Jilin, 133002, China.

Table of Contents

Materials and methods **S3**

Syntheses and characterization **S3**

Job plot for interaction of PtC_{10} with CP6A **S6**

Determination of the association constants **S7**

Fluorescence spectroscopic studies of aggregation behavior of PtC_{10} and CP6A **S8**

Critical aggregation concentrations (CACs) of free PtC_{10} and PtC_{10}⊂CP6A **S9**

TEM images of PtC_{10}⊂CP6A and DOX@PtC_{10}⊂CP6A **S10**

Schematic view of proposed PtC_{10}⊂CP6A complex **S10**

Zeta potential distribution for PtC_{10}⊂CP6A complex **S11**

Preparation of DOX@PtC_{10}⊂CP6A **S11**

***In vitro* drug release of DOX@PtC_{10}⊂CP6A** **S13**

***In vitro* cytotoxicity studies** **S14**

Cellular uptake mechanism for DOX@PtC_{10}⊂CP6A **S15**

***In vivo* antitumor efficacy** **S16**

Materials and Methods

Materials: Carboxylatopillar[6]arene (CP6A) was synthesized according to a literature method.[1] All reagents were purchased commercially and used without further purification unless otherwise noted. Minimum Eagle's medium (MEM) and Dulbecco's modified Eagle medium (DMEM) were purchased from Gibco (Thermo Fisher Scientific). Fetal bovine serum (FBS), penicillin-streptomycin and PBS were purchased from Invitrogen (Carlsbad, CA, USA). The Cell Counting Kit-8 (CCK-8) and 4',6-diamidino-2-phenylindole (DAPI) solution were purchased from Dojindo China Co. Ltd. (Shanghai, China). The HepG-2 and LO2 cells were purchased from the Cell Resource Center (Beijing, China).

Methods: ^{1}H NMR spectra were recorded using a JNM-ECA-400 spectrometer. High-resolution mass spectra (HRMS) were recorded on a Bruker Daltonics, Inc. APEXIII 7.0 TESLA FTMS instrument. UV-vis spectra were measured on a UV-2550 ultraviolet-visible spectrophotometer, Shimadzu Research Laboratory Co. Ltd. Fluorescence spectroscopic studies were carried out using a LS-55 fluorescence spectrophotometer, Perkin Elmer Co. Ltd.

Syntheses and Characterization

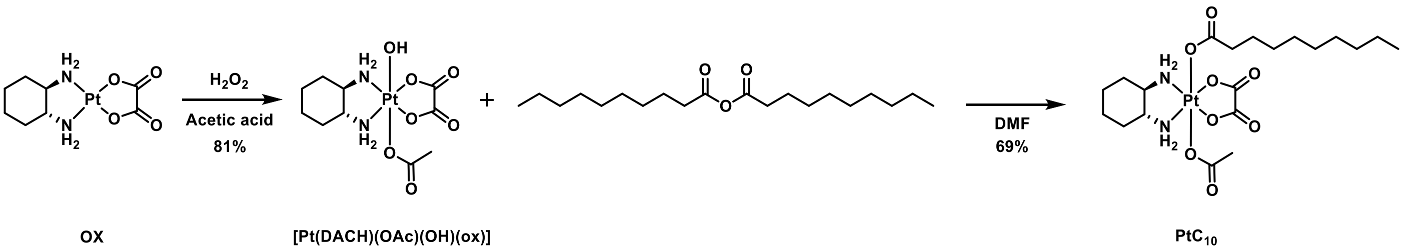

Figure S1. General synthetic route leading to PtC_{10}. Note: PtC_{10} is a new compound; it is similar to an analogous C_{16}-carbon chain reported previously.[2]

Synthesis of [Pt(DACH)(OAc)(OH)(ox)]: [Pt(DACH)(OAc)(OH)(ox)] was synthesized and purified according to previously reported procedure.[2] To a solution of OX (1.0 g, 2.5 mmol) in acetic acid (100 mL), 30% H_2O_2 (50 μL) was added under a nitrogen atmosphere. The mixture was stirred at room temperature for 6 h with the progress of the reaction being monitored by high-performance liquid chromatography (HPLC) with acetonitrile as the mobile phase. Excess acetic acid was removed by vacuum evaporation, and the resulting white solid was washed with acetone and diethyl ether before being dried under vacuum. The crude product obtained in this way was further purified by reverse-phase high-performance liquid chromatography

(RP-HPLC) using a C18 column (Waters, USA) and a gradient of acetonitrile and deionized (DI) water containing 0.1% (v/v) TFA as the mobile phase; R_T = 8.183 min. Yield: 0.96 g, 81%. ^{1}H NMR (400 MHz, D_2O) δ (ppm): 2.92, 2.91, 2.90 (m, 2H), 2.29, 2.26 (m, 2H), 2.05 (s, 3H), 1.64, 1.62 (m, 4H), 1.27, 1.24, 1.22 (m, 2H). ^{13}C NMR (100 MHz, DMSO-d_6) δ (ppm): 177.91, 163.65, 163.57, 30.82, 30.75, 23.70, 23.61, 22.72.

Synthesis of PtC_{10}: To a solution of [Pt(DACH)(OAc)(OH)(ox)] (1.0 g, 2.1 mmol) in *N,N'*-dimethylformamide (DMF), decanoic anhydride (1.0 g, 3.2 mmol) was added under a nitrogen atmosphere. The reaction mixture was stirred at 40 ºC overnight and monitored by HPLC. DMF was removed under vacuum, and the resulting light-yellow solid was washed with diethyl ether and dried under vacuum. The product was further purified by RP-HPLC using a C18 column (Waters, USA) with a gradient of acetonitrile and deionized (DI) water containing 0.1% (v/v) TFA being used as the mobile phase; R_T = 14.022 min. Yield: 0.91 g, 69%. ^{1}H NMR (400 MHz, $CDCl_3$) δ (ppm): 2.84 (m, 2H), 2.44 (m, 2H), 2.28, 2.26, 2.25 (m, 2), 1.98 (s, 3H), 1.60, 1.50 (m, 10H), 1.24 (m, 10H), 0.88, 0.88, 0.85 (t, 3H). ^{13}C NMR (100 MHz, chloroform-*d*) δ (ppm): 184.87, 182.12, 165.19, 36.52, 32.03, 29.67, 29.56, 29.48, 29.44, 25.76, 22.82, 14.28. HRMS (ESI): $C_{20}H_{37}N_2O_8Pt^+$. calcd m/z 628.6060; found m/z 628.2195.

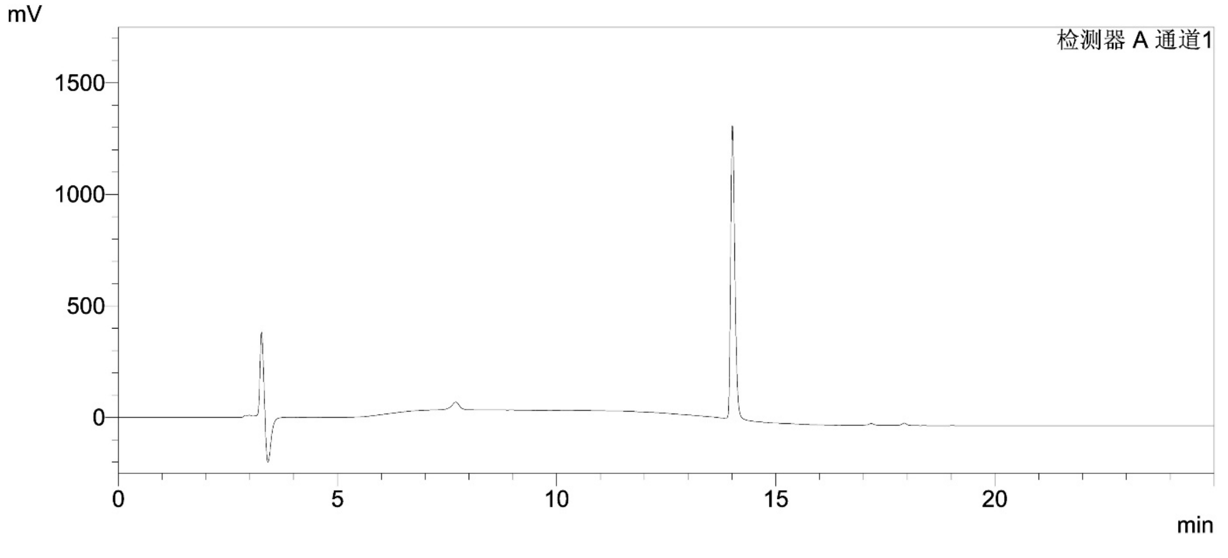

Figure S2. Reverse-phase high-performance liquid chromatogram of PtC_{10}.

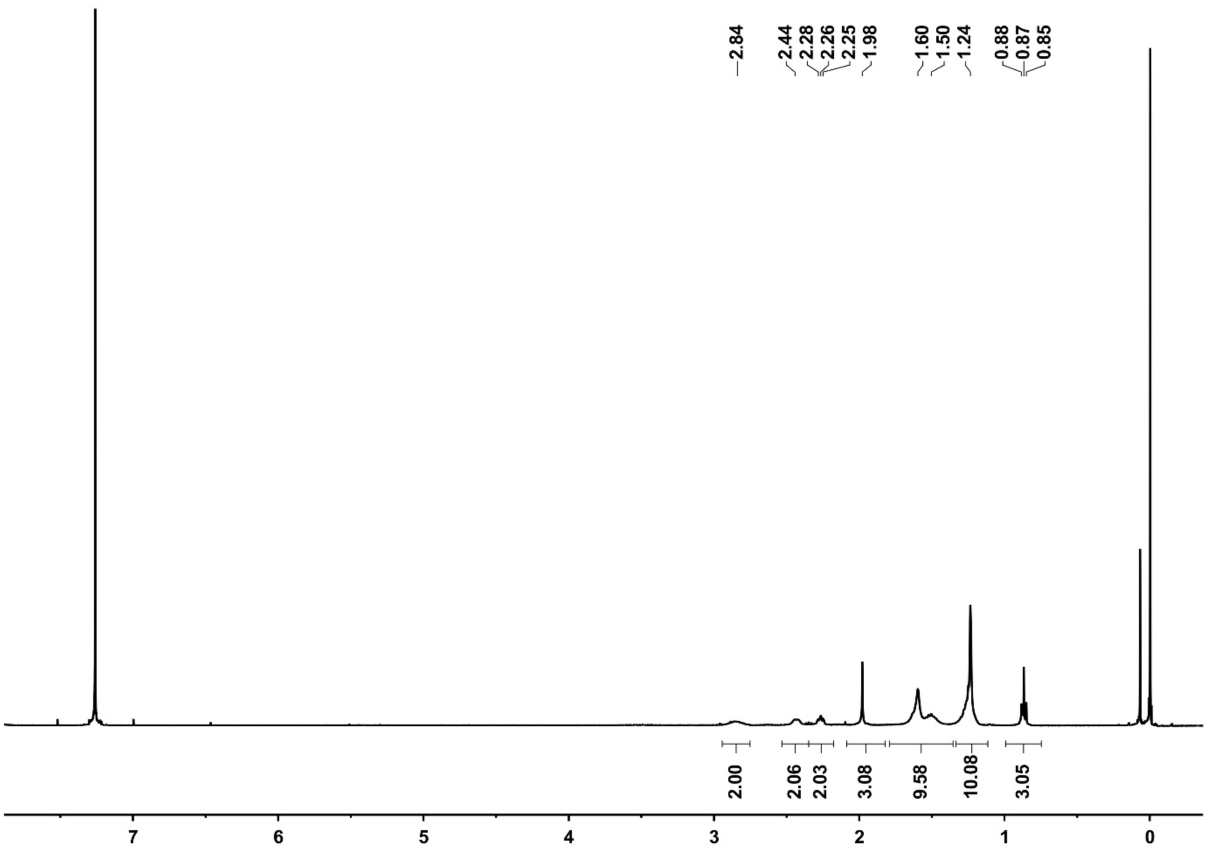

Figure S3. ^{1}H NMR spectrum (400 MHz, $CDCl_3$, 298K) of PtC_{10}.

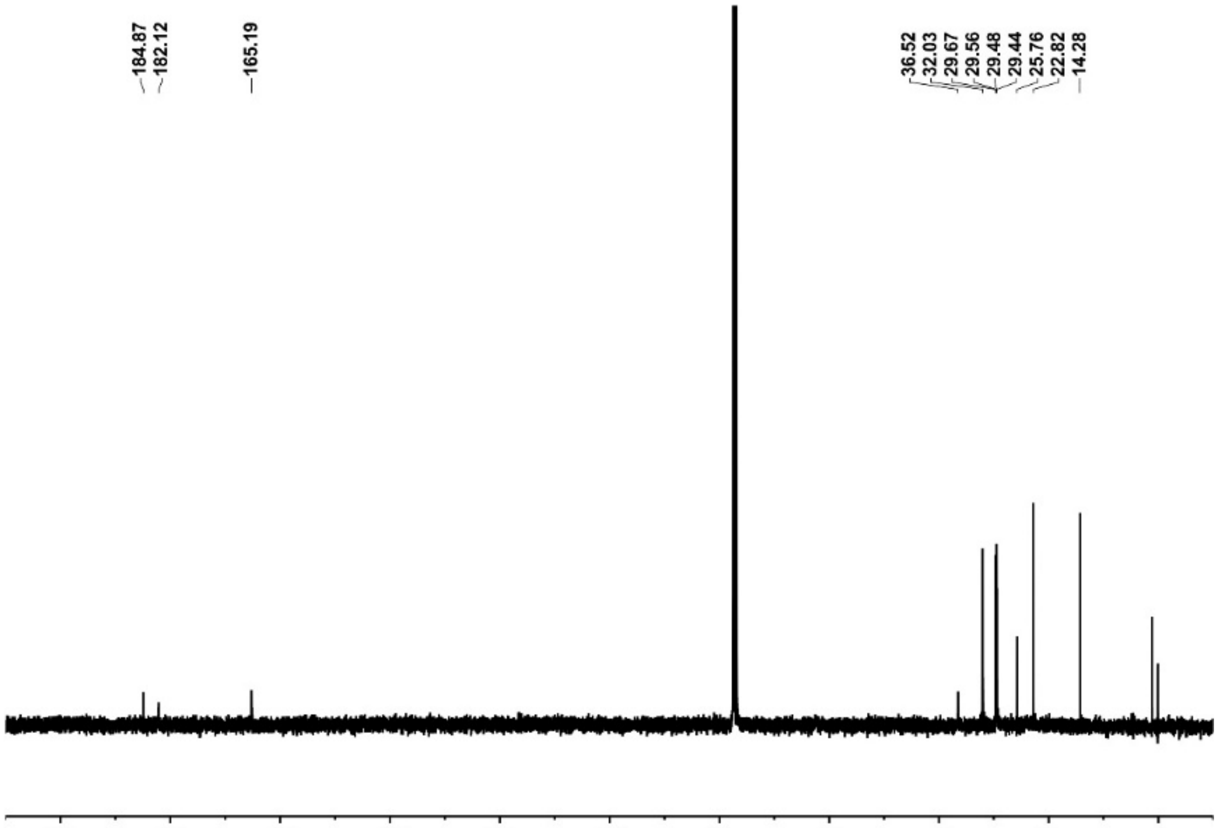

Figure S4. ^{13}C NMR spectrum (100 MHz, chloroform-*d*, 298 K) of PtC_{10}.

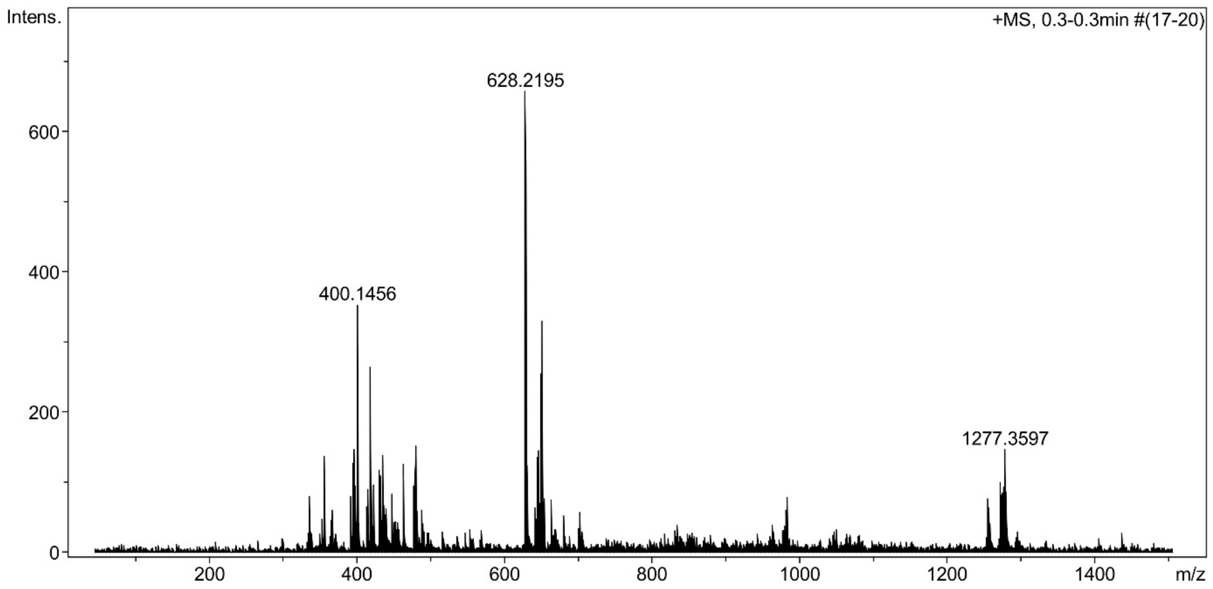

Figure S5. High-resolution mass spectrum (ESI) of PtC_{10}.

Job Plot for Interaction of PtC_{10} with CP6A

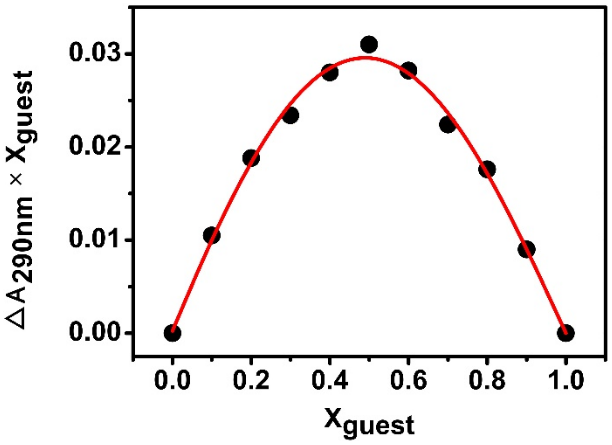

Figure S6. Job's plot for the interaction of CP6A with PtC_{10}. The maximum at a mole fraction is consistent with a proposed 1:1 stoichiometry for the complex. To construct the plot, the absorbance at 290 nm was monitored as a function of mole fraction. The study was conducted at pH 7.4 in PBS; [CP6A] + [PtC_{10}] = 1.0 × 10^{-5} M.

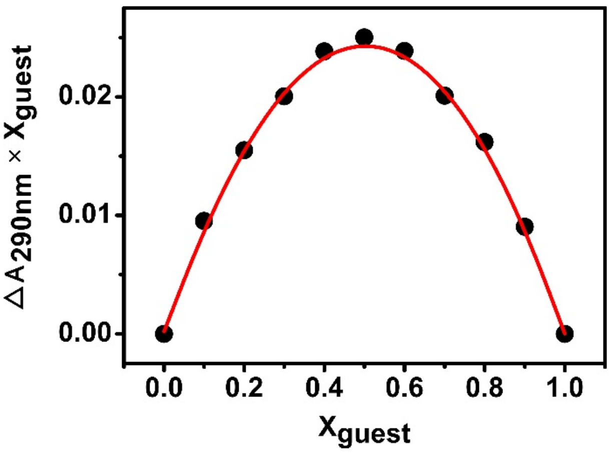

Figure S7. Job's plot for the interaction of CP6A with PtC_{10}. The maximum at a mole fraction is consistent with a proposed 1:1 stoichiometry for the complex. To construct the plot, the absorbance at 290 nm was monitored as a function of mole fraction. The study was conducted at pH 5.0 in PBS; [CP6A] + [PtC_{10}] = 1.0 × 10^{-5} M.

Determination of the association constants.

To assess quantitatively the complexation behavior of these compounds, fluorescence titrations of CP6A with PtC_{10} were performed at 298 K in phosphate buffer solutions of pH 7.4 and 5.0 per equation 1. We considered that a 1:1 host:guest complex is formed as defined by the association constant (K_a), which satisfied the law of mass action relating the equilibrium concentrations of free host ([H]), free guest ([G]) and host-guest complex ([HG]). The relationship between the total concentration of host ($[H]_0$), guest ($[G]_0$) and their equilibrium concentrations were determined in accord with the law of mass conservation (equatiosn 2-1 and 2-2). Here $[H]_0$ is the initial concentration of the guest (a known parameter that was kept constant in the titration process). Then equations 1 and 2-2 were employed to deduce equation 3. When the fluorescence titration was performed, the intensity of fluorescence (F) corresponds to the combined intensity of the host and the host-guest complex, which were described by molar fractions (equation 4). Both F_{HG} and F_H are known parameters in which F_H is the fluorescent of $[H]_0$ and F_{HG} is the fluorescent intensity when all host is complexed. Equation 5 derived from equation 2-1, 2-2, 3 and 4, explains the relationship between K_a and the variable $[G]_0$ in the fluorescence titration.

$$[\mathrm{H}] + [\mathrm{G}] \overset{K_a}{\leftrightarrow} [\mathrm{HG}]$$

$$K_a = \frac{[\mathrm{HG}]}{[\mathrm{H}][\mathrm{G}]} \qquad (1)$$

$$[\mathrm{G}] = [\mathrm{G}]_0 - [\mathrm{HG}] \qquad (2\text{-}1)$$

$$[\mathrm{H}] = [\mathrm{H}]_0 - [\mathrm{HG}] \qquad (2\text{-}2)$$

$$[\mathrm{HG}] = \frac{K_a[\mathrm{G}][\mathrm{H}]_0}{1 + K_a[\mathrm{G}]} \tag{3}$$

$$F = \frac{[\mathrm{HG}]}{[\mathrm{H}]_0} F_{\mathrm{HG}} + \frac{[\mathrm{H}]}{[\mathrm{H}]_0} F_{\mathrm{H}} \tag{4}$$

$$F = F_{\mathrm{HG}} + (F_{\mathrm{H}} - F_{\mathrm{HG}}) \frac{\left([\mathrm{H}]_0 - [\mathrm{G}]_0 - \frac{1}{K_a}\right) - \sqrt{([\mathrm{H}]_0 - [\mathrm{G}]_0 - \frac{1}{K_a})^2 + 4[\mathrm{G}]_0[\mathrm{H}]_0}}{2[\mathrm{H}]_0} \tag{5}$$

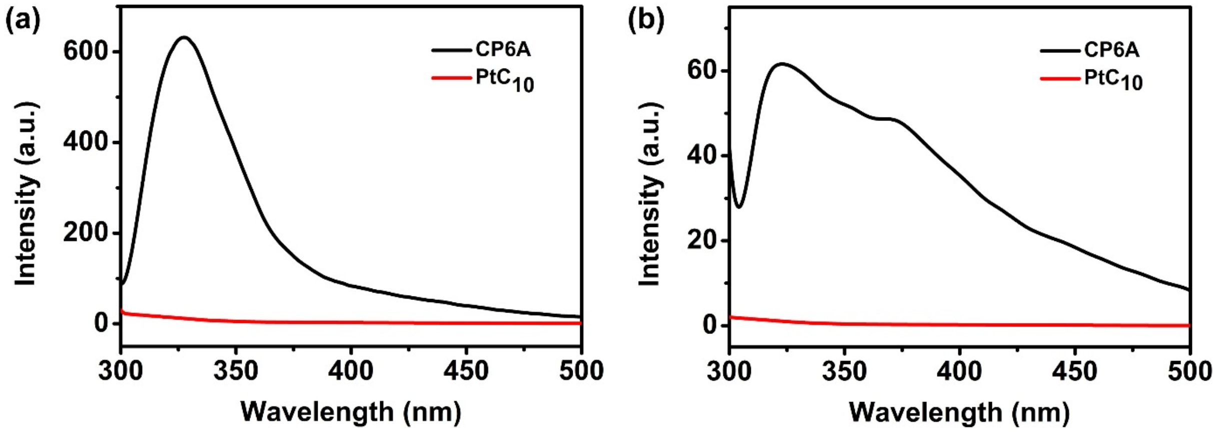

Figure S8. Fluorescence spectra of CP6A (1.0×10^{-5} M) and PtC_{10} (4.8×10^{-4} M) recorded in aqueous PBS at (a) pH 7.4 and (b) pH 5.0 at 298 K.

Fluorescence Spectroscopic Studies of Aggregation Behavior of PtC_{10} and CP6A

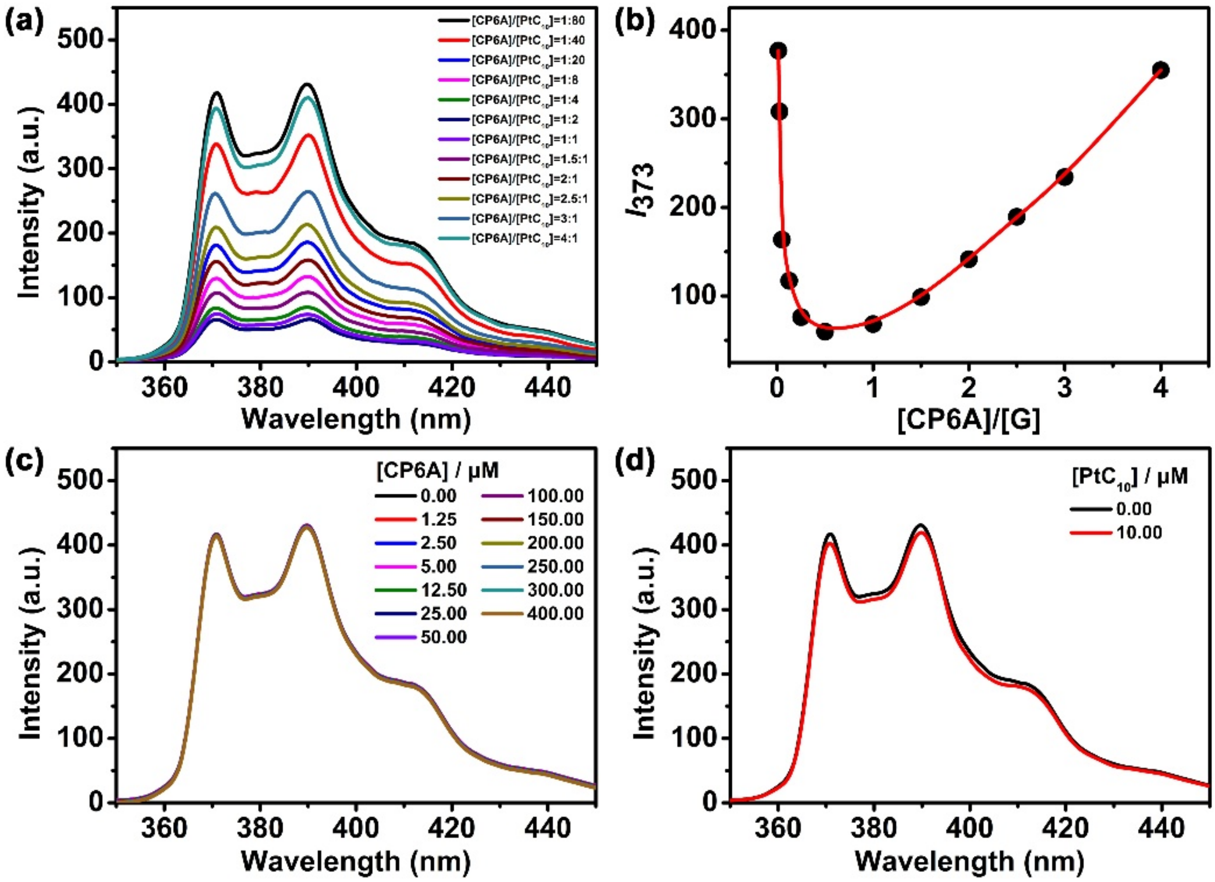

Figure S9. (a) Fluorescence emission spectra of pyrene in an aqueous solution of PtC_{10} (1.0×10^{-4} M) wherein the concentration of CP6A was gradually increased from 1.25×10^{-6} M to 4.00×10^{-4} M at 298 K. (b) Relative fluorescence intensity of pyrene plotted as a function of the molar CP6A:PtC_{10} ratio. Control groups: fluorescence emission spectra of pyrene in aqueous solutions of (c) CP6A recorded as the concentration was gradually increased from 0.00 M to 4.00×10^{-4} M and (d) PtC_{10} (0.00 M and 1.0×10^{-4} M) at 298 K.

Critical Aggregation Concentrations (CACs) of Free PtC_{10} and PtC_{10}⊂CP6A

Samples for CAC measurements were prepared by serial dilution from a 96-well plate and transferred into a 384-well black/clear microplate. The absorbance intensity (900 nm) in the samples within the microplate wells were measured using a SpectraMax® M5 plate reader (Molecular Devices, San Jose, CA, USA).[3] The intensity values were then plotted as a function of concentration. The CAC (if any) was determined from the break point in the resulting curve (cf. Figure S9).

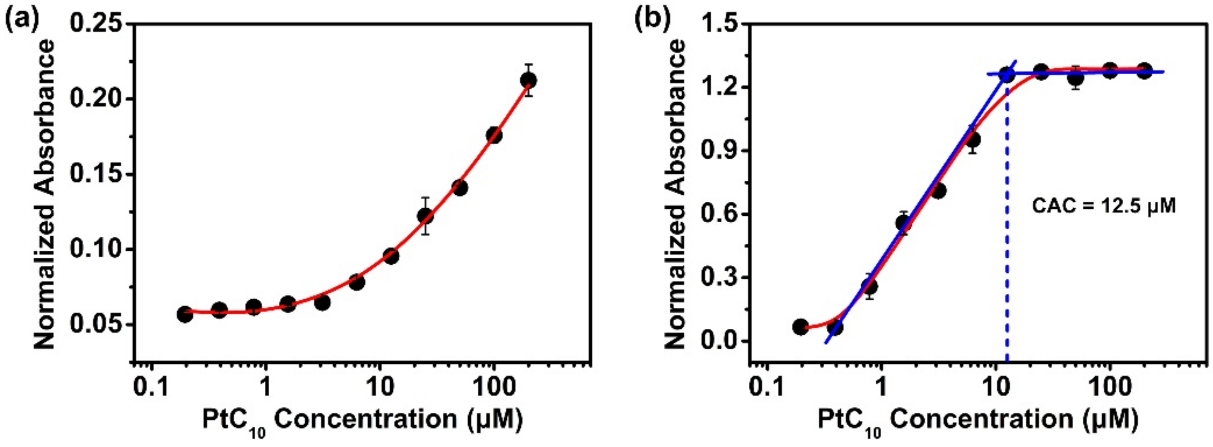

Figure S10. Normalized absorbance (900 nm) vs concentration curves for (a) free PtC_{10} and (b) PtC_{10}⊂CP6A in double-distilled water pH 6.8 (molar ratio of [PtC_{10}]/[CP6A] = 2:1).

TEM Images of PtC_{10}⊂CP6A and DOX@PtC_{10}⊂CP6A

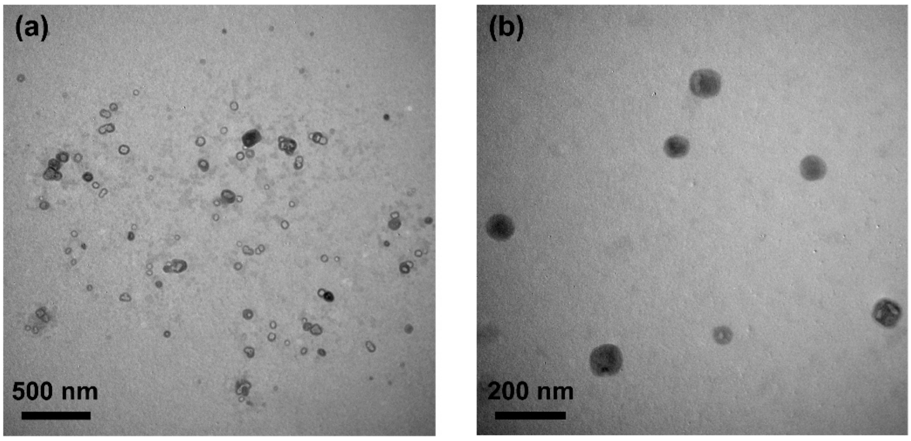

Figure S11. TEM images of (a) Vesicles self-assembled from PtC_{10}⊂CP6A and (b) DOX@PtC_{10}⊂CP6A ([CP6A] = 0.05 mM and [PtC_{10}] = 0.10 mM).

Schematic View of Proposed PtC10⊂CP6A Complex

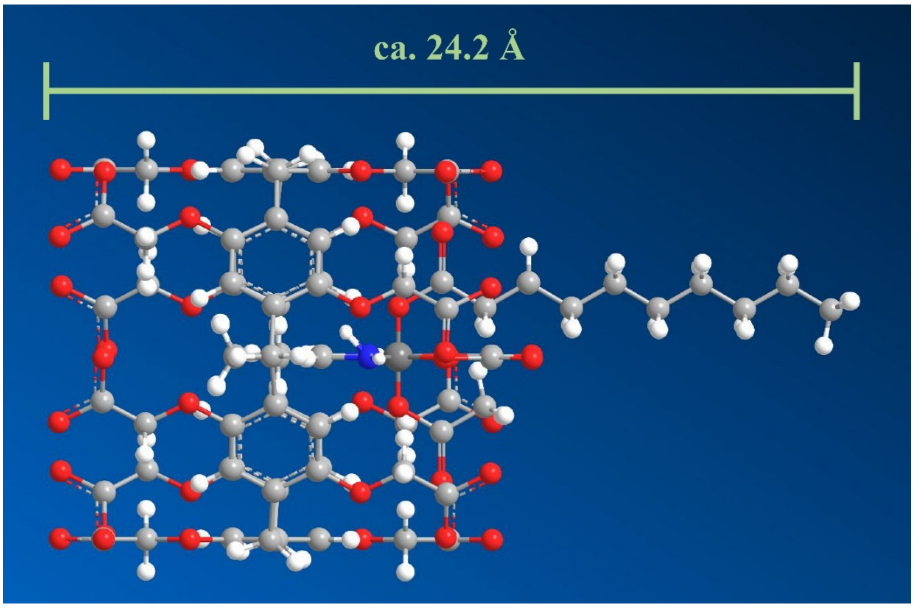

Figure S12. Model of the PtC_{10}⊂CP6A complex designed to aid in visualization (carbon atoms are gray, oxygen atoms are red, nitrogen atoms are blue, and hydrogen atoms are white). This model was constructed using Chem3D Pro 14.0.

Zeta Potential Distribution for $PtC_{10}{\subset}CP6A$ Complex

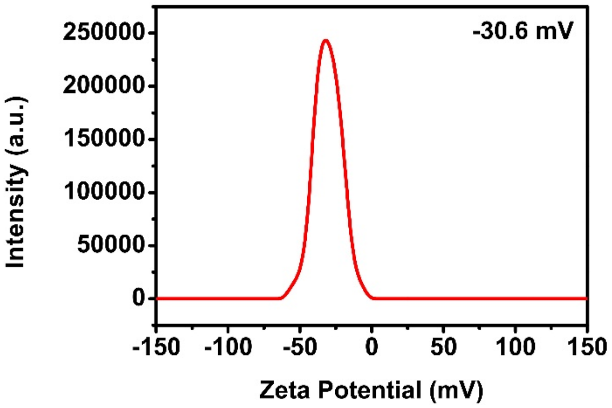

Figure S13. Zeta potential distribution for supramolecular vesicles formed from $PtC_{10}{\subset}CP6A$ ([CP6A] = 0.05 mM and [PtC_{10}] = 0.10 mM).

Preparation of DOX@$PtC_{10}{\subset}CP6A$

Doxorubicin hydrochloride (DOX)-loaded vesicles were prepared as follows: CP6A (3.26 mg, 0.20 mM) was added into double distilled water (9.8 mL) and stirred well. A solution of PtC_{10} (2.51 mg, 0.40 mM) in ethyl alcohol (0.2 mL) was added dropwise to this solution and the mixture was sonicated for 30 min. Then, DOX (2.32 mg, 0.40 mM) was added into the mixture, which was then stirred overnight. The presumed DOX@$PtC_{10}{\subset}CP6A$ constructs were purified using a dialysis bag (MW: 8000 - 14000) placed in distilled water, with the procedure being repeated several times until free DOX in the outside solution could no longer be detected by HPLC. This whole purification process was performed in the absence of light.

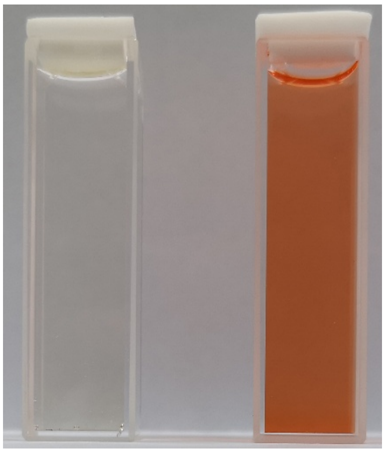

Figure S14. Images of $PtC_{10}{\subset}CP6A$ (left) and DOX@$PtC_{10}{\subset}CP6A$ (right).

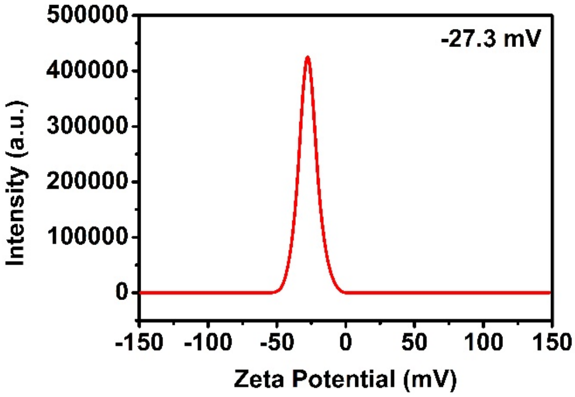

Figure S15. Zeta potential distribution for supramolecular vesicles formed from DOX@PtC_{10}⊂CP6A after dialysis.

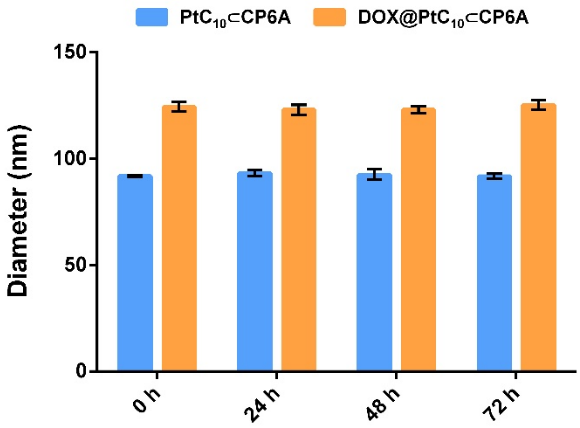

Figure S16. Diameter of PtC_{10}⊂CP6A and DOX@PtC_{10}⊂CP6A before (0 h) and after being incubated in double-distilled water for the indicated time periods as determined by DLS. No significant size change occurred over the course of 72 h, a finding interpreted in terms of PtC_{10}⊂CP6A and DOX@PtC_{10}⊂CP6A being stable under these experimental conditions (mean ± SD, n = 3).

The drug encapsulation efficiency was calculated as follows:

$$\text{Encapsulation efficiency} = \frac{W_t}{W_0} \times 100\%$$

Where W_0 is the mass of drug added, W_t is the mass of drug encapsulated in vesicles. The mass of DOX and PtC_{10} were measured by HPLC and calculated relative to a standard calibration curve over the 0.078 to 40.00 µM concentration range in double distilled water. By diluting five-fold with 1% Triton X-100 and two-fold with ethyl alcohol, nearly 100% release of DOX and PtC_{10} from the drug-loaded vesicles could be achieved.

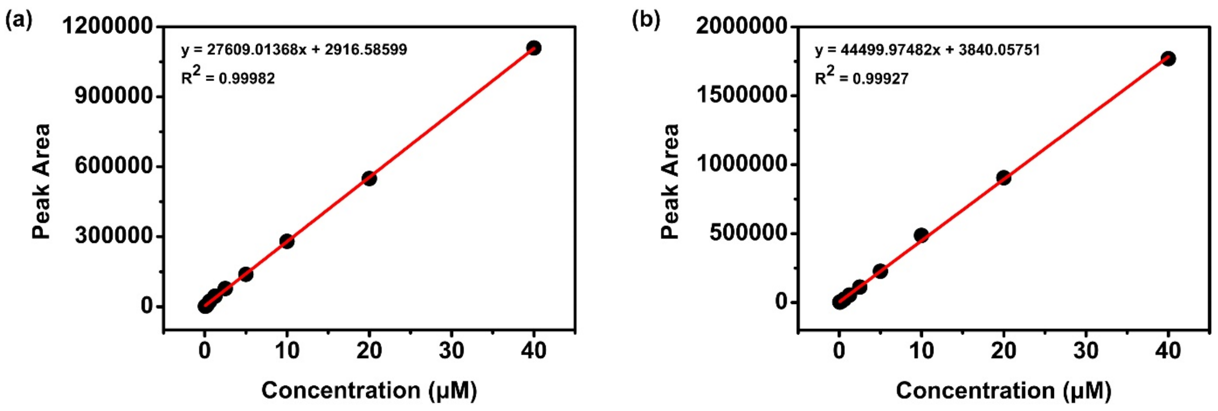

Figure S17. Calibration curves obtained by HPLC and used for calculating (a) the DOX concentration and (b) the PtC_{10} concentration in the release studies described in the main text.

In Vitro Drug Release of DOX@PtC_{10}⊂CP6A

The release of DOX and PtC_{10} *in vitro* was tested using dialysis bags (molecular weight cutoff: 8000 - 14000). Phosphate buffer solution (0.1 mM) with different pH (5.0 and 7.4) was used as release medium. Free DOX or DOX@PtC_{10}⊂CP6A (1 mL of a corresponding aqueous solutions) was added to the dialysis bag and immersed in 10 mL of the release medium under stirring (200 rpm, preheated to 37 ± 1 °C). 1 mL dialysate samples were withdrawn and replaced with an equal volume of fresh media at predetermined intervals. The concentration of DOX in the medium was determined by HPLC. The cumulative amount of DOX released was calculated as follows:

$$\text{Cumulative release} = \left(\frac{V_0 C_0 + V_s \sum_1^{n-1} C_t}{m_0}\right) \times 100\%$$

Where m_0 is the total mass of drug in free DOX solution or DOX@PtC_{10}⊂CP6A, V_0 is the initial medium volume, V_s is the sampling volume, C_0 and C_t are the drug concentrations, t and n are the sampling times. All assays were performed in triplicate in the absence of light.

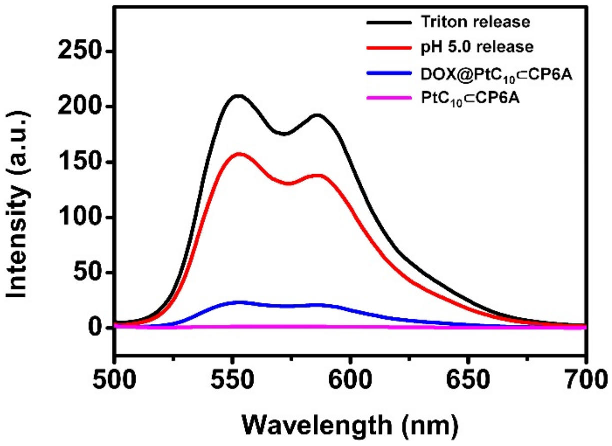

Figure S18. Fluorescence spectra of PtC_{10}⊂CP6A, DOX@PtC_{10}⊂CP6A and the spectrum of DOX@PtC_{10}⊂CP6A recorded after presumed pH 5.0- and Triton X-100-mediated release.

In Vitro Cytotoxicity Studies

The relative cytotoxicity of CP6A against two cell lines was assessed *in vitro* using CCK-8 according to the manufacturer's instructions. Human liver hepatocellular carcinoma cells (HepG-2) were seeded into 96-well plates at a density of 8000 cells/well in 100 μL of MEM supplemented with 10% FBS, 1% penicillin, and 1% streptomycin. Likewise, human normal liver cells (LO2) were seeded into 96-well plates at a density of 8000 cells/well in 100 μL of complete DMEM supplemented with 10% FBS, 1% penicillin, and 1% streptomycin and cultured for 24 h in 5% CO_2 at 37 °C. CP6A was dissolved in PBS and then diluted to the required concentration. It was then added to the cell-containing wells which were further incubated at 37 °C under 5% CO_2 for 72 h. Subsequently, 10 μL of CCK-8 was added into each well and incubated for another 0.5 h. The plates were then measured at 450 nm using a SpectraMax® M5 plate reader (Molecular Devices, San Jose, CA, USA). All experiments were carried out five independent times. Cell viability was calculated as follows:

$$\text{Cell Viability} = \frac{\text{OD}_{\text{test}} - \text{OD}_{\text{blank}}}{\text{OD}_{\text{control}} - \text{OD}_{\text{blank}}} \times 100\%$$

Where OD_{blank} is the optical density of blank well (medium and CCK-8 reagent), OD_{test} is the optical density of the test group and $OD_{control}$ is the optical density of the control group.

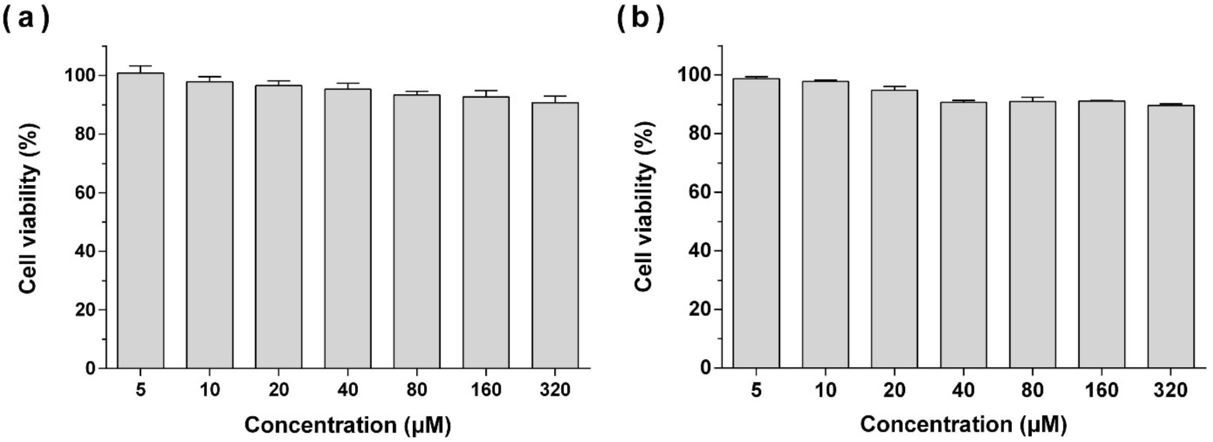

Figure S19. Relative cell viabilities of (a) HepG-2 and (b) LO2 cells after incubation for 72 h with CP6A at the indicated concentrations. Cell death was then measured by using a CCK-8 assay (mean ± SD, *n* = 5).

Table S1. IC_{50} values (μM) of PtC_{10}, OX, DOX, OX+DOX, DOX@PtC_{10}⊂CP6A and CI values of OX+DOX, DOX@PtC_{10}⊂CP6A against the HepG-2 and LO2 cell lines.

Formulation	IC_{50} (μM)		CI [b]	
	HepG-2	LO2	HepG-2	LO2
PtC_{10}	5.25	9.48		
OX	3.47	8.61		
DOX	0.34	0.38		
OX+DOX	0.16 [a]	0.20 [a]	0.62	0.60
DOX@PtC_{10}⊂CP6A	0.17 [a]	0.18 [a]	0.61	0.54

[a]The IC_{50} value of OX+DOX and DOX@PtC_{10}⊂CP6A groups represent for the concentration of DOX. [b]The combination index, CI, is defined as follows: $\mathrm{CI} = \frac{D_1}{D_{m1}} + \frac{D_2}{D_{m2}}$, where D_1 and D_2 represent the concentration values of drug 1 and drug 2 when used in combination to inhibit half the cell growth, respectively, and D_{m1} and D_{m2} represent the half-maximum inhibitory concentration values of each drug alone.

Cellular Uptake Mechanism for DOX@PtC_{10}⊂CP6A

HepG-2 cells were seeded into 6-well plates at a density of 200,000 cells/well in 1.5 mL of MEM supplemented with 10% FBS, 1% penicillin, and 1% streptomycin and cultured for 24 h in 5% CO_2 at 37 °C. The cells were then treated with several internalization inhibitors for 0.5 h: 0.1 M 5-(*N*-ethyl-*N*-isopropyl) amiloride (EIPA) for inhibiting macropinocytosis; 0.4 M sucrose, and 0.05 M chlorpromazine (CP) for clathrin-coated vesicle formation; 0.05 M ammonium chloride (AC) for lysosome function. Cells were also incubated at 4 °C to minimize all energy-dependent uptake pathways. The cells were then rinsed with PBS before DOX@PtC_{10}⊂CP6A was added and the cells incubated for 4 h. After 4 h, the cells were harvested and washed three times with cold PBS and resuspended in 500 μL PBS. DOX fluorescence was detected by flow cytometery. Cells incubated without inhibitor were used as a negative control.

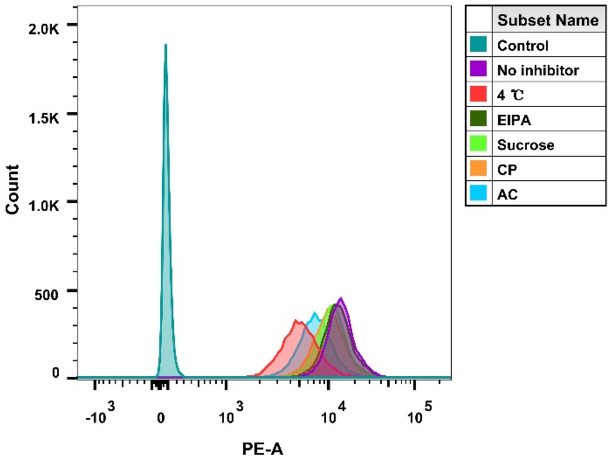

Figure S20. FACS assays of cellular uptake pathways of DOX@PtC$_{10}$⊂CP6A (containing 10 μM DOX) in HepG-2 cells using the indicated endocytosis inhibitors. Note: we prepared DOX@PtC$_{10}$⊂CP6A through decreasing the nanoparticles concentration, the administration dosage of which was still high than CAC value.

In Vivo Antitumor Efficacy

Six-week-old male BALB/c nude mice (~20 g body weight) were purchased from the Beijing Experimental Animal Center (Beijing, China). A total of 1 × 10^6 HepG-2 cells in 200 μL of saline were inoculated subcutaneously into the right dorsal flanks of the mice. The mice were normally fed after inoculation for 1 week. In total, 30 mice were randomly divided into five groups. The control group (5% glucose to mimic what is used in the clinic for OX administration) and the other groups containing 5% glucose and either OX (8.90 mg·kg^{-1}), DOX group (4.00 mg·kg^{-1}), OX+DOX group (8.90 mg·kg^{-1} OX + 4.00 mg·kg^{-1} DOX, molar ratio = 3.25) and DOX@PtC$_{10}$⊂CP6A group (18.29 mg·kg^{-1} CP6A + 14.06 mg·kg^{-1} PtC$_{10}$ + 4.00 mg·kg^{-1} DOX) were subject to intravenous (i.v.) injection in mice at different time points (on days 1, 3, 5, 7, and 9). After treatment, the tumor volumes and the weights of the mice were recorded at different time points. On day 11, the mice were sacrificed and the tumors were separated from the animals and weighed. All experimental procedures were conducted in accordance with the Guide for the Care and Use of Laboratory Animals of the AAALAC, and were approved by the Animal Care and Use Committee of the National Beijing Center for Drug Safety Evaluation and Research. Best efforts were made to minimize the number of animals used and their suffering. Tumor volumes were calculated as follows:

$$\text{Tumor Volume } (mm^3) = \frac{1}{2} \times a \times b^2$$

In this equation, *a* and *b* represent the largest and smallest tumor diameters, respectively.

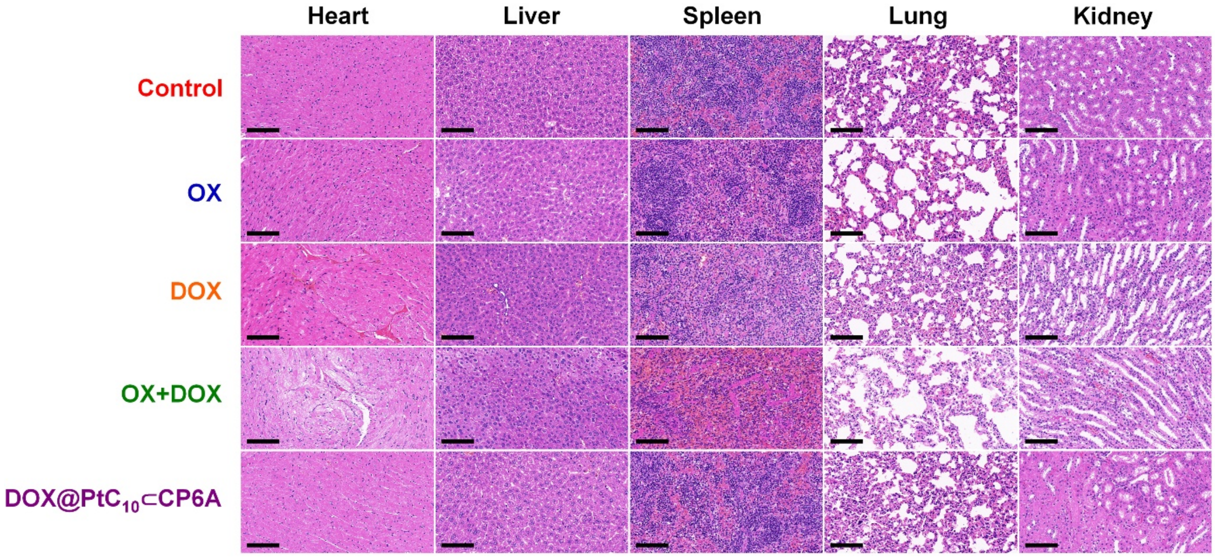

Figure S21. Histopathologic analysis of major organs (heart, liver, spleen, lung and kidneys) stained with H&E 10 days after treatment with 5% glucose solution (control), OX, DOX, OX+DOX and DOX@PtC_{10}⊂CP6A also in glucose (scale bar: 100 μm).

References

1 C. Li, X. Shu, J. Li, S. Chen, K. Han, M. Xu, B. Hu, Y. Yu, X. Jia, *J. Org, Chem.,* 2011, **76**, 8458-8465.

2 A. A. Ammar, R. Raveendran, D. Gibson, T. Nassar, S. Benita, *J. Med. Chem.,* 2016, **59**, 9035-9046.

3 L. Cai, M. Gochin, K. Liu, *Chem. Commun.,* 2011, **47**, 5527-5529.
